# The Creaming of Short Doughs and Its Impact on the Quality Attributes of Rotary-Molded Biscuits

**DOI:** 10.3390/foods10030621

**Published:** 2021-03-15

**Authors:** M. Teresa Molina, Sandra M. Vaz, Pedro Bouchon

**Affiliations:** 1 Departamento de Ingeniería Química y Bioprocesos, Escuela de Ingeniería, Pontificia Universidad Católica de Chile, Avda, Vicuña Mackenna 4860, Macul, Santiago de Chile 7820436, Chile; mtmolin1@uc.cl; 2 Nestlé Development Centre, Camino a Melipilla 15300, Maipú, Santiago de Chile 9260075, Chile; sandra.muntz.vaz@gmail.com

**Keywords:** rotary-molded biscuits, granulated sucrose, powdered sucrose, low-shear mixer, high-shear mixer, creaming stability, X-ray micro-CT, sensory profiling, image analysis

## Abstract

Scant attention has been given to understanding the impact of creaming stability on the final structure of semi-sweet biscuits, an aspect that has traditionally concerned the biscuit industry. Accordingly, the aim of this study was to analyze the influence of the creaming phase stability on the quality attributes of rotary-molded biscuits. Doughs were formulated with 10.2% of fat (wet basis) and 16.3% of sucrose (w.b.), using two sucrose particle sizes, which were either added directly or after dilution in water at different concentrations. Additionally, the creaming phase was prepared using either a low-shear or a high-shear mixer. The results show that an aqueous-phase migration occurred when the creaming was blended in a low-shear mixer, when using either powdered sucrose or granular sucrose diluted in water at a high concentration. The phase separation was inhibited with the high-shear mixer, which provided a stable creaming. Notwithstanding the variation in creaming stability, no differences were observed in hardness, aeration, sweetness, color and noise intensity. Additionally, the micro-CT analysis revealed that biscuits had a similar microstructure (air porosity and thickness of biscuit walls) when they were prepared with either an unstable or a stable creaming phase. Consequently, creaming stability does not seem to affect the structure and the most relevant sensory attributes of rotary-molded biscuits under this set of experimental conditions, which are representative of those used by the industry for this product category.

## 1. Introduction

Biscuits are classified according to their formulation and the way they are produced, giving rise to the following biscuit categories: deposited, wafer, laminated, sugar-snap, rotary-molded and wire-cut [1]. Sugar-snap, rotary-molded and wire-cut biscuits are obtained from a short dough, which differentiate them from the other classes as they have a higher amount of sugar and fat and a lower amount of water [2]. In general, short doughs contain soft flour (9–11% total weight of protein), 17–33% of sugar (dough weight, w.b.) and 9–21% of fat (dough weight, w.b.) [1]. However, there is a group of rotary-molded doughs that contain lower amounts of sugar (10–20% dough weight, w.b.) and fat (6–12% dough weight, w.b.), and their effect on biscuit making has received less attention.

The mixing process is a critical stage of biscuit manufacture as it influences gluten development and hence the rheological properties of the short dough [3]. Accordingly, horizontal mixers with a low rotation speed are usually used during this stage. The geometry of mixers may include single or double sigma blades or a shaftless mixer blade. Industrial mixers operate between 30 and 70 rpm, whereas the speed of laboratory mixers can reach up to 200 rpm [4]. The mixing process is generally divided into two steps in order to constrain the water–flour interaction as much as possible so that the formation of the gluten network is restricted: these are referred to as the creaming (or cream-up) and dough-up stages. During creaming preparation, fat, sucrose, water, leavening agents and surfactants are blended together to produce a homogeneous, emulsified and aerated phase. During the dough-up phase, the flour and the rest of the leavening agents are added to the creaming phase, using a low mixing speed and in the shortest possible time, in order to restrict the mechanical work and gluten development hindrance [5,6,7]. At the end of mixing, a short dough is obtained, which is described as a suspension of proteins and starch granules in a continuous liquid–sugar solution, where lipids are emulsified and constitute the disperse phase [8]. Sugars can be added to the dough formulation as crystals or as a sugar solution. Several studies have focused on the impact of sugar dissolution and its particle size on the quality attributes of sugar-snap and wire-cut biscuits, which have shown to be crucial parameters during biscuit processing as they influence dough expansion during baking [9,10,11,12,13]. It has been suggested that fine sucrose particles dissolve faster in water during baking, promoting the lateral spread of the dough that subsequently allows a larger and thinner biscuit to be obtained compared to the effect of coarser crystals, which produce shorter and thicker biscuits as a consequence of their slower dissolution in water during heating [12]. Both the sugar crystal size and the concentration of the sugar solution may provide a better understanding of their concomitant influence on the final attributes of rotary-molded biscuits, which contain lower amounts of sugar than wire-cut or sugar-snap biscuits. However, to date no studies have addressed this effect on this biscuit category.

Furthermore, it has been stated that the creaming phase should be homogeneous and emulsified to prevent flour hydration during the dough-up phase [2,5,6,14]. However, limited attention has been given to understanding whether an unstable creaming phase would actually have a negative effect on the final attributes of biscuits. An unstable creaming phase could potentially affect dough processing as the water or the sugar solution may influence the functionality of flour polymers. Although the addition of a sugar solution delays the hydration of flour compared to just water, this phenomenon is not inhibited [13]. Kweon et al. [15] declared that the hydration of gluten proteins with a sugar solution is thermodynamically more favorable than hydration with just water, but the kinetics of gluten development are retarded when increasing the sugar concentration. Different mixing methods have been examined to assess the impact of flour hydration on the quality attributes of biscuits. Manohar et al. [3] observed that the elastic recovery and cohesiveness of rotary-molded dough increased when all the ingredients were blended at once during mixing (all-in-one method), instead of when using two stages (with a creaming step), attributing this behavior to the gluten development due to the higher water availability in the system. Regarding the mixing time and the mixing speed, most of the studies focused on the effect of these parameters during the dough-up phase [16,17]. However, it is still necessary to understand the impact of mixing parameters on the stability of the creaming phase and how this condition may affect the quality attributes of biscuits. High-shear mixers offer higher rotation speeds than horizontal mixers, ranging from 1000 to 25,000 rpm [18], but they are not commonly used during creaming elaboration. These types of mixers may provide a stable and emulsified creaming phase due to their high mechanical energy input, which increases the interfacial area of the disperse phase and consequently may influence rotary-molded dough performance.

Accordingly, the aim of this study was to determine whether an unstable or stable creaming phase may modify the quality attributes (i.e., dimensions, microstructure, hardness and sensory profiling) of rotary-molded biscuits when using different sucrose particle sizes and sugar concentrations when adding a sugar solution.

## 2. Materials and Methods

### 2.1. Ingredients of Rotary-Molded Biscuits

The dough was prepared using commercial soft-wheat flour (Molinera San Cristobal, Santiago, Chile) [composition: 70% starch, 13.9% moisture, 10% proteins, 3.6% total fiber, 2% lipids, 0.5% ash; 55% water absorption; 8.1% dry gluten; 4.3% damaged starch], palm oil (Teamfoods, Santiago, Chile), granulated or powdered sucrose (Iansa, Santiago, Chile), soy lecithin (Cargill, Santiago, Chile), ammonium bicarbonate (Basf, NJ, USA), sodium bicarbonate (Andimex S.A., Santiago, Chile), monocalcium phosphate (Blumos, Santiago, Chile) and salt (K + S Chile S.A., Santiago, Chile).

### 2.2. Biscuit Formulation and Preparation

The standard dough was formulated by mixing refined flour (65.2%, d.b.), sucrose (19.7%, d.b.), fat (12.3%, d.b.), leavening agents (2.5%, d.b.), soy lecithin (0.2%, d.b.), salt (0.1%, d.b.) and water (10.2% d.b.). Two sucrose particle sizes were used: granulated (GS, D90 = 978 µm) and powdered sucrose (PS, D90 = 98 µm). The amount of water (150 g, 10.2% d.b.) was divided into two fractions. Fifty grams was used to dissolve the leavening agents and the salt in all formulations. The remaining portion (100 g) was used to prepare four sugar solutions, based on sugar saturation (ST), which are referred to as 0%ST, 50%ST, 100%ST and >100%ST, in order to study the effect of the addition of a sugar solution on the stability of the creaming phase. These solutions were prepared considering the solubility of sucrose at 20 °C (i.e., 204 g of sucrose per 100 g of water). 0%ST refers to pure water. In this case, 288 g of sucrose (19.7%, d.b.) and 100 g of water were separately added to the mix. The 50%ST solution means that 102 g of sugar was dissolved in 100 g of water, and the remaining fraction (186 g) was added as crystals to the formulation. The 100%ST solution means that 204 g of sugar was dissolved in 100 g of water, and the remaining 84 g was added as crystals to the mix. The >100%ST solution was an oversaturated sugar solution, which was obtained by dissolving 288 g of sucrose in 100 g of water by heating and stirring it at 70 °C, in order to ensure the complete dissolution of sucrose crystals. This solution was finally cooled down and added to the dough preparation at 25 ± 1 °C.

**Figure 1:**
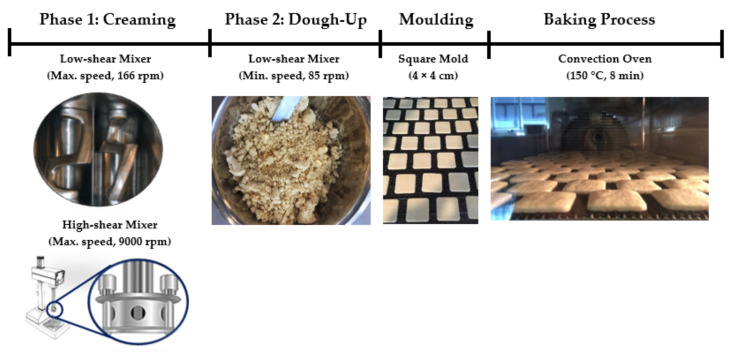
Elaboration process of rotary-molded biscuits.

The dough was prepared in two steps, including a creaming and a dough phase, as illustrated in Figure 1. During the creaming step the fat, sugar crystals and/or sugar solution, sodium bicarbonate, ammonium bicarbonate, lecithin, salt and water were mixed for 5.5 min. This phase was prepared using two different mixers, in order to examine the effect of creaming stability on the quality attributes of rotary-molded biscuits. To do so, either a horizontal mixer at the highest nominal speed (166 rpm average, Laboratory Z-blade mixer, Morton-Mixers, United Kingdom) or a vertical high-shear mixer at the highest nominal speed (9000 rpm, SL2T, Silverson, United Kingdom) was used. Thereafter, the flour and monocalcium phosphate were added to the creaming phase and the mix was blended in a horizontal mixer for 60 s at minimum speed (85 rpm) to obtain the dough. The dough was then molded into square molds (4 × 4 cm) using a minilab rotary moulder (RTech Limited, Merseyside, United Kingdom), and the thickness of the dough was 2.5 mm. Seventy-eight dough pieces per tray were baked at 150 °C for 8 min using a convection oven (SALVA, Gipuzkoa, Spain) until the biscuits reached a final moisture content of 2.0 ± 0.2% (w.b.).

### 2.3. Aqueous-Phase Migration from the Creaming Phase

The stability of the creaming phase was analyzed by transferring the cream to a separating funnel, where it was left for phase separation until equilibrium (1 h at 21 ± 1 °C). The aqueous phase was then collected and weighted. The aqueous-phase migration was reported as the percentage of the aqueous-phase weight over the cream weight [19].

### 2.4. Firmness of the Creaming Phase

The firmness of the creaming phase was determined at 21 ± 1 °C using a penetration test in a TA.XT Texture Analyzer (5 kg loadcell, Stable Microsystems, Surrey, United Kingdom), employing a conical TTC spreadability rig (HDP/SR). Prior to the analysis, the probe height was calibrated to ensure a starting point at the same height for each test (25.0 mm). The creaming phase was filled into the female cone, and the male cone was lowered up to 23 mm at a test speed of 3 mm/s. Five measurements per batch were taken. The force (N) at the maximum penetration depth was taken as a texture parameter of firmness [20].

### 2.5. Properties of Rotary-Molded Biscuits

The moisture content of biscuits was analyzed thermogravimetrically using a HB43 halogen moisture analyzer (METTLER TOLEDO, Lutz FL, USA), where 5 g of ground sample was heated at 125 °C with a switch-off criterion 3. The dimensions of the biscuits (i.e., length, width and thickness) were measured with a digital vernier caliper (0–150 mm, Würth, Guelph, Canada), and thirty biscuits per batch were evaluated. The maximum breaking force of biscuits was measured after two days, using a TA.XT Texture Analyzer (5 kg loadcell, Stable Microsystems, Surrey, United Kingdom). Prior to the analysis, the samples were hermetically sealed using a trilaminate film, PET/BOPP Met/PE, and were then stored at 20 ± 1 °C. Twenty biscuits per batch were analyzed employing a three-point bending test (HDP/3PB probe) with a support span of 36 mm, using a test speed of 1 mm/s. The maximum breaking force (N) upon compression was used as a texture descriptor of biscuit hardness [21].

### 2.6. X-ray Micro-Computed Tomography (X-ray µCT)

The microstructure of rotary-molded biscuits was analyzed using a Skyscan 1272 X-ray micro-computed tomography system v.1.1.7 (Bruker Corp., Kontich, Belgium), where the X-ray source operated at a voltage of 45 kV and a current of 222 µA. Images were acquired using an exposure time of 480 ms, with a rotation step of 0.2° over an interval of 0°–360°, and two frames averaging. Three samples were scanned for each condition.

Around 1800 projection images were obtained from the image acquisition, which were then further processed using reconstruction software NRecon v. 1.7.3 (Bruker Corp., Billerica, MA, USA) to obtain 2D cross-sectional images (resolution 2016 × 1344 pixels, voxel size of 10.7 µm^3^). During the reconstruction step, thermal correction (X/Y alignment with a reference scan), misalignment compensation (post-alignment), smoothing (1, using Gaussian kernel = 2), ring artifacts reduction (=5) and beam hardening correction (=45%) were set to obtain a good quality of the reconstructed images. The reconstructed images were processed and analyzed using CTAn software v. 1.17 (Bruker Corp., Belgium), according to the following steps: (i) selection of a volume of interest (VOI); (ii) removal of residual noise; (iii) definition of a region of interest (ROI) prior to segmentation; (iv) segmentation of biscuit components (biscuit matrix and air pores); and (v) 3D quantification using the structure thickness, which enables a 3D representation of the size distributions of biscuit components to be obtained [22].

### 2.7. Sensory Analysis

The sensory attributes of biscuits (i.e., hardness, aeration level, noise intensity, grittiness, sweetness, color and thickness) were analyzed with 10 trained panelists using the monadic profiling technique. The definition of each sensory attribute is presented in Table 1. The intensity of the sensory attributes was evaluated in two assessments, as recommended by Meyners et al. [23] and Moser et al. [24]. An eleven-point hedonic scale was used during the evaluation, from 0 to 10, where the intensity ranges were defined as follows: 0.1 to 2 (weak); 2.1 to 4 (slightly weak); 4.1 to 6 (moderate); 6.1 to 8 (slightly strong); and 8.1 to 10 (strong). During the test, the panelists inserted the samples into their mouths and the sensory attributes were evaluated during biscuit chewing using FIZZ software v. 5.2 (Biosystemes, France). After consuming each sample, panelists were instructed to rinse their mouths by drinking water and eating some plain crackers, as proposed by Kohyama et al. [25], and to rest for 15 min before taking the following sample test. A total of five sessions were required to conduct the whole evaluation.

### 2.8. Statistical Analysis

The experimental data were acquired in triplicate (3 batches per condition) and they were analyzed with R (R Foundation for Statistical Computing, Vienna, Austria), version 3.6.1. A one-way Welch ANOVA was applied when *n* ≥ 30 (biscuit dimensions and maximum breaking force), in order to analyze whether the treatment means differed from each other. This test is used when homoscedasticity cannot be assumed but normally distributed data are required, where the central limit theorem approximation is good enough if the sample size is greater than 30 [26]. The pairwise post hoc analysis was performed using the Games–Howell test at 95% confidence, which does not assume homoscedasticity and equal sample sizes [27]. However, when *n* < 30, the bootstrap method, introduced by Efron [28], was used as it does not make any assumption on the underlying population distribution (i.e., a non-parametric approach) [29]. In particular, the analysis performed in this study did not assume that the data came from a normal distribution and instead it fully accepted that the population distribution was unknown. The bootstrap method uses sampling with replacement on the existing data to recover the unknown population distribution of the parameters of interest (mean in this case) and to estimate confidence intervals. To estimate this effect, one thousand replicates were created, and 95% confidence intervals were obtained to test differences between the means of the different formulations.

## 3. Results and Discussion

In order to determine whether the stability of the creaming phase has an impact on the quality and the sensory attributes of rotary-molded biscuits, stable and unstable creams were prepared by modifying the mixer configuration, the sucrose particle size and the concentration of the sucrose solution. Accordingly, the characterization of the creaming phase and the associated results are described in the following sections.

### 3.1. Characterization of the Creaming Phase

**Figure 2:**
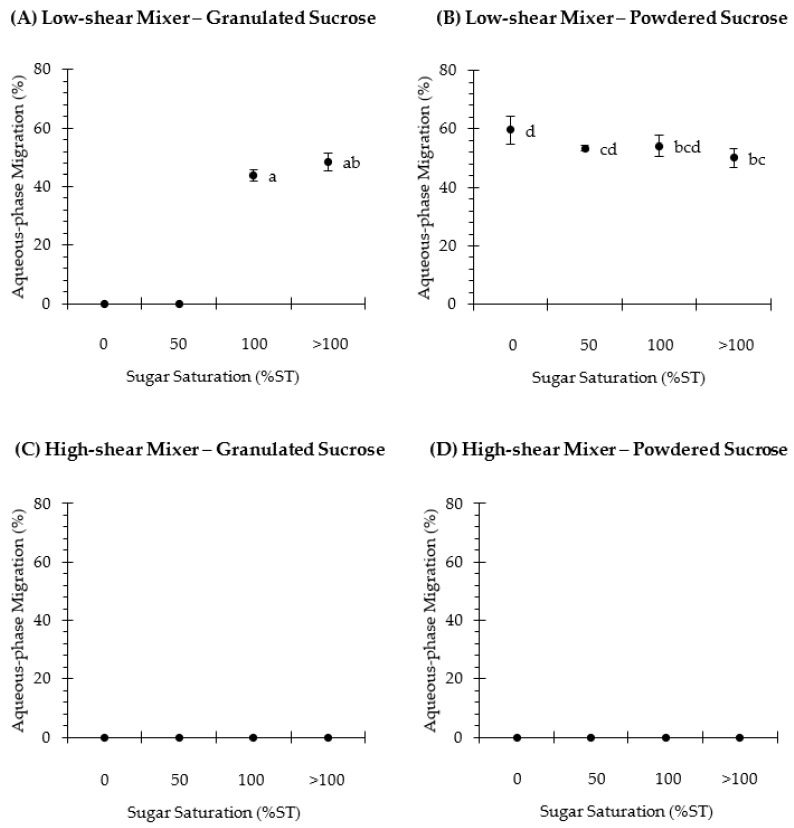
Aqueous-phase migration (%) of the creaming phase, which was elaborated using granulated (**A**,**C**) or powdered (**B**,**D**) sucrose and was then subjected to low-shear (**A**,**B**) or high-shear (**C**,**D**) mixing. Additionally, sucrose was dissolved in water to obtain the following sugar solutions: 0%ST, 50%ST, 100%ST or >100%ST. Data are mean ± confidence intervals at 95% (*n* = 3). Different scripts denote significant differences (*p* < 0.05).

Figure 2 shows the aqueous-phase migration, which was obtained as explained in Section 2.3. It can be observed that when the cream was prepared using a low-shear mixer (166 rpm) (Figure 2A,B), the sucrose particle size and the concentration of sugar in the sucrose solution affected the stability of the cream. While granulated sucrose allowed a stable cream to be obtained when adding either the 0%ST or the 50%ST solution, the cream was unstable with powdered sucrose at all concentrations, and ~53–59% of the aqueous-phase migrated from the cream. During mixing of the creaming phase, two phenomena occur simultaneously: (i) sugar dissolution, and (ii) emulsifier adsorption at the interface between the oil and water. Sugar dissolution depends on the sucrose particle size. Accordingly, powdered sucrose will probably dissolve faster than granulated sucrose because its surface-area-to-volume ratio is approximately four times higher (28.1, 1/mm) than the value for granulated sucrose (6.7, 1/mm) [30]. Moreover, in oil-in-water emulsions, stabilization of oil droplets requires mechanical energy to disrupt coarse droplets and an effective adsorption of the emulsifier, which is able to reduce the interfacial tension at the interface, strongly bind to the interface and protect the oil droplets against flocculation and coalescence [31]. Due to the slow rotational speed that may be achieved with the horizontal mixer, it is suggested that a stable creaming phase is difficult to obtain due to the insufficient disruptive force to fragment large oil droplets into smaller ones, as referred to by McClements et al. [32]. On the other hand, according to Figure 2C,D, it can be observed that the aqueous-phase migration was inhibited when using a high-shear mixer during creaming preparation, as neither the sucrose particle size nor the sugar solution affected the stability. The high-shear action of the mixer, and its disintegrating head geometry, most probably enhanced the homogenization of the water (or sugar solution) and the oil phase. The energy applied when using a high rotation speed (9000 rpm, nominal) helps to increase the interfacial area of the dispersant phase (into droplets) and improve the distribution of the surfactant [33], so that a stable creaming phase may be obtained.

**Figure 3:**
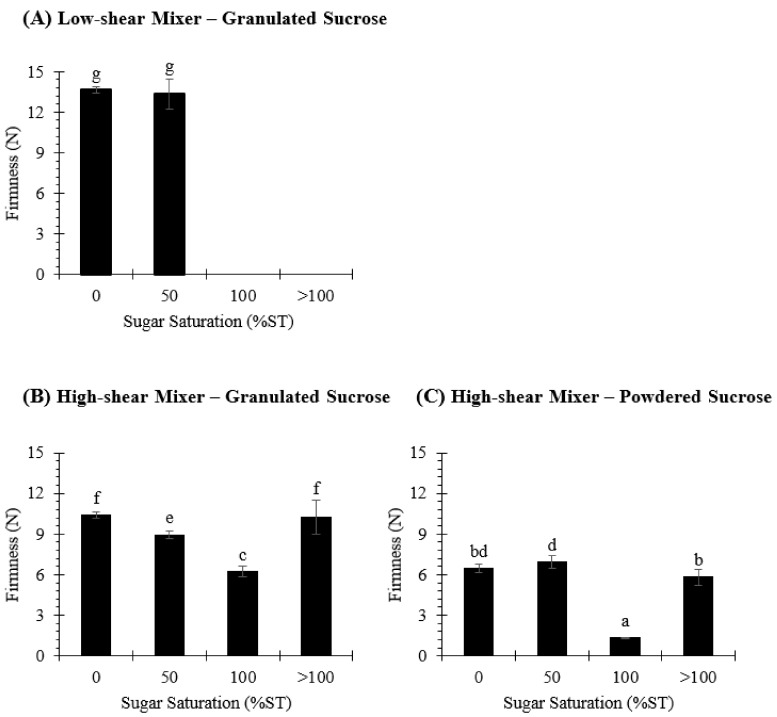
Firmness (N) of the creaming phase, which was elaborated using granulated (**A**,**B**) or powdered (**C**) sucrose and was then subjected to low-shear (**A**) or high-shear (**B**,**C**) mixing. Additionally, sucrose was dissolved in water to obtain the following sugar solutions: 0%ST, 50%ST, 100%ST or >100%ST. Data are means ± confidence intervals at 95%. Different scripts denote significant differences (*p* < 0.05).

The firmness of the stable creaming phases was measured using a spreadability test, in order to understand whether this variable may influence the way biscuit dough spreads during baking and, consequently, whether it may be related to the final dimensions of the biscuits. As shown in Figure 3, the highest degree of firmness (~13 N) was obtained in creamings formulated with granulated sucrose and prepared using a low-shear mixer, and no significant differences (*p* > 0.05) were observed when water (0%ST) or an unsaturated sugar solution (50%ST) was used (Figure 3A). The use of a high-shear mixer significantly decreased (*p* < 0.05) the firmness of the cream (Figure 3B,C), and the sucrose particle size affected this parameter. As a matter of fact, solutions prepared with powdered sucrose were consistently less firm than those prepared with granulated sucrose. The shearing action and the mixing time are possibly not enough to transform all the coarser sugar crystals (granulated sucrose) into finer particles with a similar distribution to that from powdered sucrose, leading to greater firmness. Interestingly, an increase in the amount of sugar dissolved in water tended to decrease the firmness of the cream. This could be related to the extra volume of the aqueous phase that is obtained as a result of sugar dissolution, as suggested by Ghiasi and RC [34]. However, when a >100%ST sugar solution was used, the firmness of the creaming phase increased, becoming similar to the value observed in creams prepared with a 0%ST sugar solution. This result does not follow the trend that was observed in the other samples and was expected to obtain a similar or even lower firmness value to that found in creams prepared with a sugar solution at 100% ST. This behavior could be related to sucrose crystallization induction in a supersaturated solution, which is catalyzed by the agitation energy input and the presence of small sugar crystals acting as secondary nuclei, as explained by Hartel [35]. These factors could affect the sucrose–water interaction [36], thus modifying the structure of the creaming phase to a system similar to that obtained using a 0%ST sugar solution. Notwithstanding the foregoing, further studies are necessary to better understand the effect of sugar dissolution and the saturation point on the rheology and the microstructure of the stabilized creaming phase of rotary-molded biscuits.

### 3.2. Dimensions of Rotary-Molded Biscuits

The dimensions of rotary-molded biscuits were measured following baking. Results are reported in Table 2. Horizontal dimensions are linked to the lateral spread of the dough during baking from its original size (40 × 40 mm^2^), while the thickness is related to the vertical expansion of the dough. It can be observed that the length and width were smaller (*p* < 0.05) in biscuits for which creaming was prepared using the high-shear mixer compared to those subjected to low-shear mixing, with only one exception in both dimensions (granulated sucrose at >100%ST sugar solution). For instance, the length and width of samples elaborated with granulated sucrose and a sugar solution of 0%ST were 39.53 and 39.88 cm for biscuits prepared using a high-shear mixer and 39.95 and 40.30 cm for those when a low-shear mixer was used, respectively. Nevertheless, the differences did not exceed 0.8 mm in length (2%) and 0.25 mm in width (1%), meaning that the variation was considered rather negligible. These results may indicate that the differences observed in the firmness of the creaming did not modify the lateral spread of the dough during baking because minor changes were obtained in the horizontal dimensions of rotary-molded biscuits. In turn, the thickness was similar between samples, as no differences (*p* > 0.05) were found when comparing the effect of each mixer. When evaluating biscuits that were prepared using low-shear mixing, it was determined that the sucrose particle size did not modify (*p* > 0.05) the length of biscuits, and the width was, at most, 0.39 mm smaller (*p* < 0.05) in biscuits with powdered sucrose. A similar behavior was observed in biscuits prepared with high-shear mixing, where minor differences (<0.45 mm) were detected among samples. Furthermore, the concentration of the sucrose solution slightly affected the dimensions of the biscuits. However, no clear pattern was observed, and the differences were not larger than 0.73 mm (1.8%) for the horizontal dimensions and 0.2 mm (3.7%) for the thickness. Previous studies have shown that the horizontal dimensions of sugar-snap and wire-cut biscuits are greater in products elaborated with finer sucrose crystals or when using a sugar solution [7,12,37]. In particular, Kweon et al. [12] observed that biscuits prepared with coarser sugar crystals (>500 µm) had smaller diameters than those prepared with finer crystals (<500 µm), a result that was attributed to the slower dissolution of coarse crystals during mixing and baking. Overall, results presented in Table 2 show that the sucrose particle size and the percentage of sugar dissolved in water practically do not modify the horizontal dimensions of rotary-molded biscuits, as opposed to what has been found in wire-cut and sugar-snap biscuits.

### 3.3. Hardness of Rotary-Molded Biscuits

**Figure 4:**
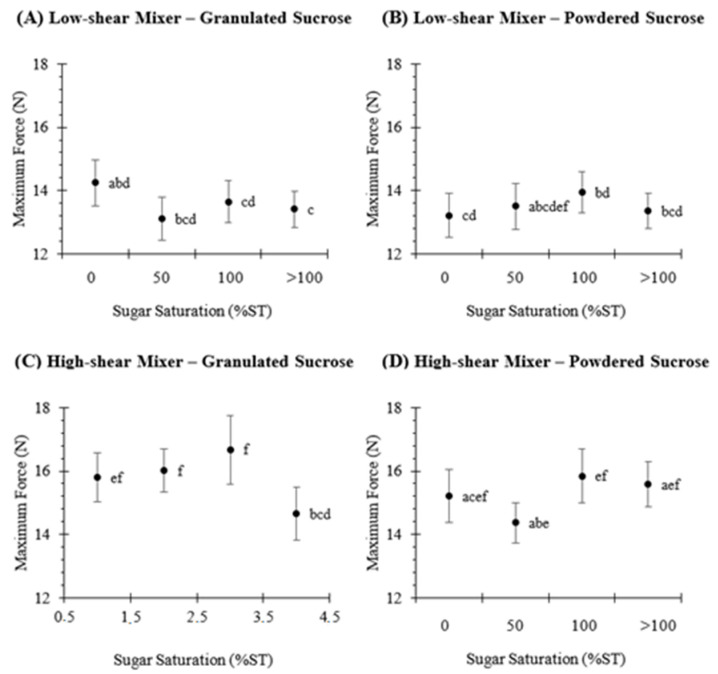
Maximum breaking force (N) of rotary-molded biscuits, obtained by texture analyzer. Biscuits were elaborated using granulated (**A**,**C**) or powdered (**B**,**D**) sucrose. Sugar was dissolved in water to obtain the following sugar solutions: 0%ST, 50%ST, 100%ST or >100%ST. Further, the creaming phase was subjected to low-shear (**A**,**B**) or high-shear (**C**,**D**) mixing. Data are means ± confidence intervals at 95%. Different scripts denote significant differences (*p* < 0.05).

In order to understand the effect of creaming stability on the texture of rotary-molded biscuits, the maximum breaking force was measured using a texture analyzer (Figure 4). The maximum breaking force in all biscuits ranged between 13.1 and 16.7 N. Moreover, biscuits whose creaming was prepared using a low-shear mixer (Figure 4A,B) were between 1 and 3 newtons less hard (*p* < 0.05) than biscuits prepared with a high-shear mixer (Figure 4C,D). For instance, in biscuits prepared with granulated sucrose and water (0%ST sugar solution), this value was ~14.2 and 15.8 N in samples prepared using a low-shear (Figure 4A) and a high-shear mixer (Figure 4C), respectively. Furthermore, the concentration of the sucrose solution did not impact (*p* > 0.05) the maximum breaking force except in biscuits prepared with granulated sucrose at the >100%ST sugar solution. However, the difference was lower than 0.83 (5.8%) and 1.13 N (7.1%) compared to samples prepared with water (0%ST), when using low- and high-shear mixing, respectively (Figure 4A,C). In addition, the sugar crystal size did not influence the maximum breaking force (*p* > 0.05) in biscuits prepared with low-shear mixing. In turn, the sucrose particle size only influenced the maximum force at 50%ST in samples prepared with a high-shear mixer, where the value was only 1.65 N higher in biscuits prepared with granulated sucrose.

**Figure 5:**
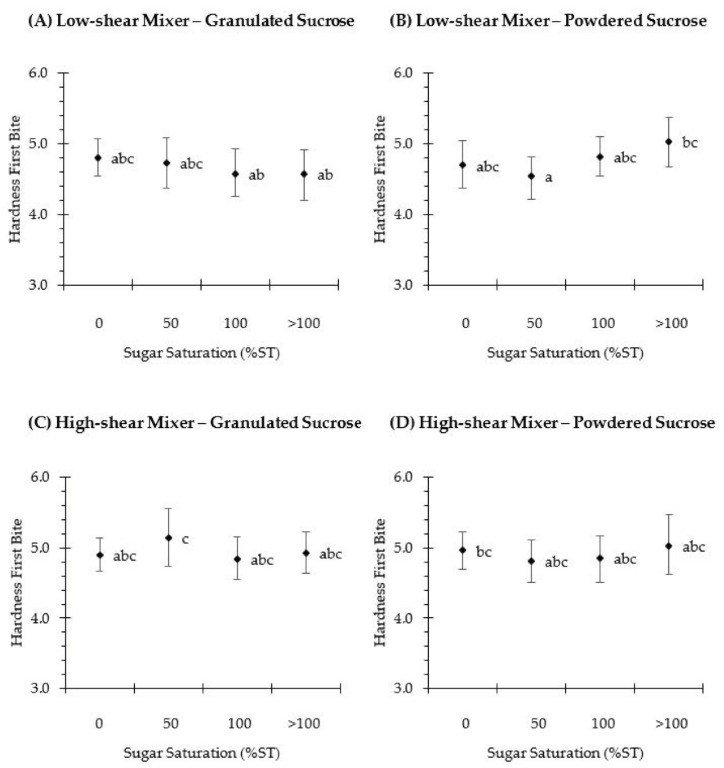
Hardness at the first bite of rotary-molded biscuits, obtained by a trainee sensory panel. Biscuits were elaborated using granulated (**A**,**C**) or powdered (**B**,**D**) sucrose. Sugar was dissolved in water to obtain the following sugar solutions: 0%ST, 50%ST, 100%ST or >100%ST. Further, the creaming phase was subjected to low-shear (**A**,**B**) or high-shear (**C**,**D**) mixing. Data are means ± confidence intervals at 95%. Different scripts denote significant differences (*p* < 0.05).

A sensory analysis was also performed to examine the perception of biscuit hardness by a trained panel. Additionally, the maximum breaking force and the hardness at the first bite were compared to ascertain whether the force increment within the range of 1 to 3 N could actually be perceived by the panelists. As can be seen in Figure 5, all biscuits were scored between 4.6 and 5.1, which corresponds to a moderate hardness in the intensity range. The concentration of the sugar solution did not affect the hardness at the first bite except in biscuits prepared with powdered sucrose using low-shear mixing at the 50%ST and >100%ST sugar solutions (Figure 5B). Despite this difference, the results were in the same range within the sensory scale, and a variation of 0.27 between them may be considered negligible. Additionally, the differences determined with the texture analyzer (Figure 4) were not perceived by the sensory panelists. Regarding the sugar crystal size, this variable did not impact the hardness at the first bite (*p* > 0.05) (Figure 5), and the differences obtained instrumentally were not detected by the panelists. Furthermore, they did not detect any variation in biscuit hardness when its corresponding creaming phase was prepared using a low-shear or a high-shear mixer (Figure 5A,C and Figure 5B,D). Kim et al. [38] prepared biscuits with different amounts of sugar to investigate the correlation between the hardness response obtained by three instrumental tests and by a trained sensory panel. When replicates of biscuits with the same amount of sugar were analyzed (for instance, 13.4% w.b.), and even though the same level of hardness was detected by the panelists, the bending test showed differences in hardness of up to 4.6 N between samples. Similar results were obtained by Yılmaz et al. [39], who also found that a difference of 5 N (measured using a texture analyzer) between two samples was not detected by the trained panel. Accordingly, it seems reasonable that a difference of 3 N measured instrumentally would not be perceived by trained panelists and even less by consumers.

### 3.4. Additional Sensory Attributes of Rotary-Molded Biscuits

**Figure 6:**
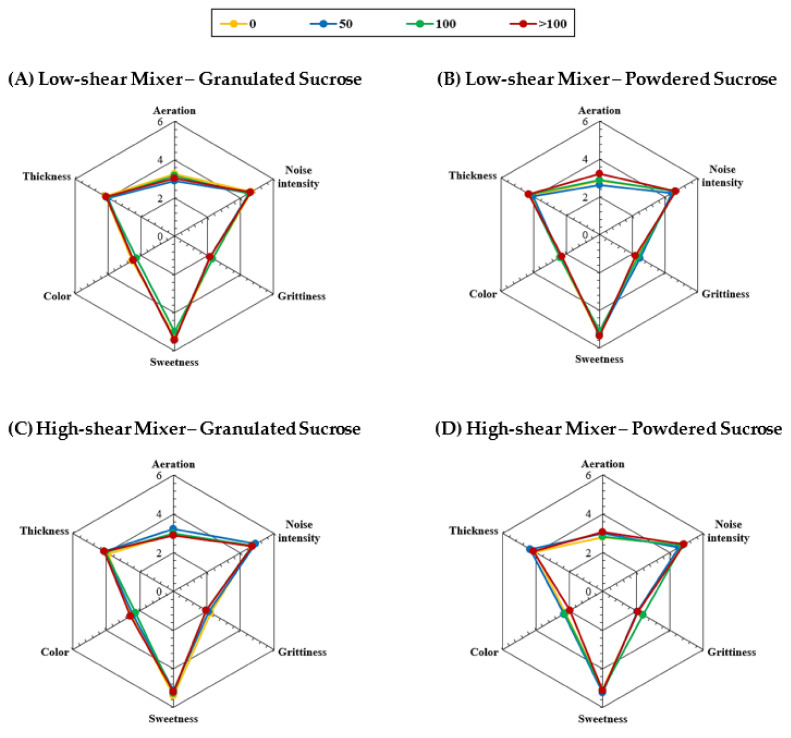
Sensory attributes (i.e., aeration, noise intensity, grittiness, sweetness, color, thickness) of rotary-molded biscuits. Biscuits were elaborated using granulated (**A**,**C**) or powdered (**B**,**D**) sucrose. Sugar was dissolved in water to obtain the following sugar solutions: 0%ST (yellow), 50%ST (blue), 100%ST (green) or >100%ST (red). Further, the creaming phase was subjected to low-shear (**A**,**B**) or high-shear (**C**,**D**) mixing. Data (*n* = 20) are expressed as observed mean.

To better understand the effect of creaming stability and sucrose (particle size, sugar in solution) on the sensory profiling of rotary-molded biscuits, the aeration, noise intensity, grittiness, sweetness, color and thickness were determined using a monadic profile with a trained sensory panel. Figure 6 displays a 2D radar graph, where the sensory attributes of different biscuits are compared. In addition, Table 3 shows the statistical analysis and confidence intervals at 95% of these sensory attributes. It can be observed that all samples had similar sensory profiling (Figure 6), where the mean score range for each attribute was as follows: 2.7 to 3.3 for aeration, which corresponds to a slightly weak intensity; 4.4 to 4.9 for noise intensity, which is a moderate intensity; 2.0 to 2.5 for grittiness (slightly weak intensity), which is associated with the presence of sugar crystals, indicating that they were not perceived by the panelists; 5.0 to 5.4 for sweetness, which corresponds to a moderate intensity; 2.0 to 2.6 for color (slightly weak intensity), which is related to a light yellowish color; and 3.9 to 4.3 for thickness. Regarding the sucrose particle size, no significant differences (*p* > 0.05) were observed in any of the sensory attributes when biscuits were prepared with a low-shear mixer (Table 3). The same trend was observed in samples prepared using a high-shear mixer, apart from color, where a slight but minor difference was observed between samples prepared with the >100%ST sugar solution. Furthermore, at equal sugar crystal sizes, the type of mixer did not influence the sensory attributes (*p* > 0.05) at any concentration of the sugar solution. Accordingly, these results also show that a stable or unstable creaming phase does not produce any variation in the sensory attributes of rotary-molded biscuits.

Regarding the concentration of the sucrose solution used for creaming preparation, it was only in four attributes that some significant differences between samples (p < 0.05) were found (bold marks in Table 3): aeration in “B” samples with 50%ST and >100%ST sugar solutions; sweetness in “A” samples with 50%ST and 100%ST sugar solutions; color in “D” samples with 50%ST and >100%ST sugar solutions; and thickness in “C” samples with 0%ST and >100%ST sugar solutions. Despite this, the intensity range was similar between each pair, and no specific relationship was found among these samples. Furthermore, the sweetness and the grittiness were similar between samples, indicating that these attributes were not perceived as being different when sucrose was incorporated as crystals or pre-dissolved in water. Analyzing the biscuit formulation, the sucrose/added water ratio was 1.9. In most of the samples, part of the sugar crystals was pre-dissolved prior to mixing, although a fraction also remained as crystals. Bearing in mind that the solubility of sucrose increases with temperature, for instance, from 2.07 $\frac{\;g\;sucrose}{g\;water}$ at 25 °C to 4.76 $\frac{\;g\;sucrose}{g\;water}$ at 100 °C [40], it is suggested that the remaining part of the sugar crystals can be completely dissolved during baking when the sucrose/added water ratio is well below the solubility of sucrose at 100 °C.

### 3.5. Microstructure of Rotary-Molded Biscuits

**Figure 7:**
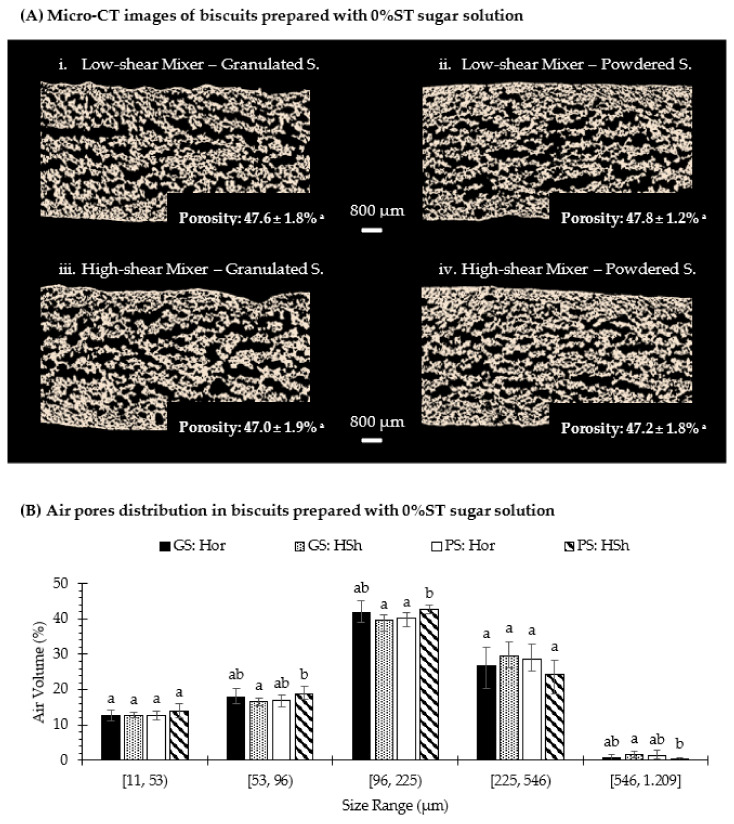
2D X-Y cross-sectional images (by X-ray µCT) of biscuits (**A**) and structure thickness distribution of air pores inside the biscuit structure (**B**), which were formulated with a sugar solution at 0%ST, using granulated (GS) or powdered (PS) sucrose, and the creaming phase was subjected to low-shear (Hor) or high-shear (HSh) mixing. Porosity is means ± standard deviation (*n* = 3). Data from air pores distribution are means ± confidence intervals at 95%. Different scripts in each range interval denote significant differences (*p* < 0.05).

The mechanical properties of biscuits are related to their microstructure and affect the perception of their sensory attributes during chewing [41]. To better understand the effect of creaming stability on the microstructure of biscuits, the air porosity and the size distribution of air pores were analyzed (3D quantification), as shown in Figure 7. Only biscuits prepared with water (0%ST sugar solution) were selected as previous results showed that the concentration of the sugar solution did not alter either the maximum breaking force or the sensory attributes of the biscuits. Figure 7A presents the 2D cross-sectional images of biscuits prepared with an unstable or stable creaming phase, where the air pores are represented in black and the biscuit matrix in beige white. The air porosity (%) was measured from 3D micro-CT images and corresponded to the percentage of the volume of voxels of air pores over the total volume of voxels. All samples had a similar air porosity (p > 0.05), which is consistent with that obtained in the aeration attribute by sensory analysis, and their mean values were 47.6% (Figure 7A(i)), 47.8% (Figure 7A(ii)), 47.0% (Figure 7A(iii)) and 47.2% (Figure 7A(iv)). Besides this information, the thickness distribution of the structure of air pores was also examined through micro-CT image analysis, as shown in Figure 7B. Around 30% of air pores were smaller than 96 µm, ~70% of them were less than 225 µm and about 98% were located below 546 µm, which is in agreement with Pareyt et al. [42], who found that a sugar-snap biscuit with 17.6% sugar (w.b.) had most of its air pores below 637.26 µm. Moreover, at equal sucrose particle sizes, the type of mixer did not produce significant differences (*p* > 0.05) in the air pores size distribution of rotary-molded biscuits, except for biscuits with powdered sucrose in the range of (96, 225) µm. The difference was not pronounced because biscuits prepared with a high-shear mixer (stable creaming) had 42.6% of air pores located in that range compared to 40.2% for biscuits prepared with a low-shear mixer (unstable creaming).

**Figure 8:**
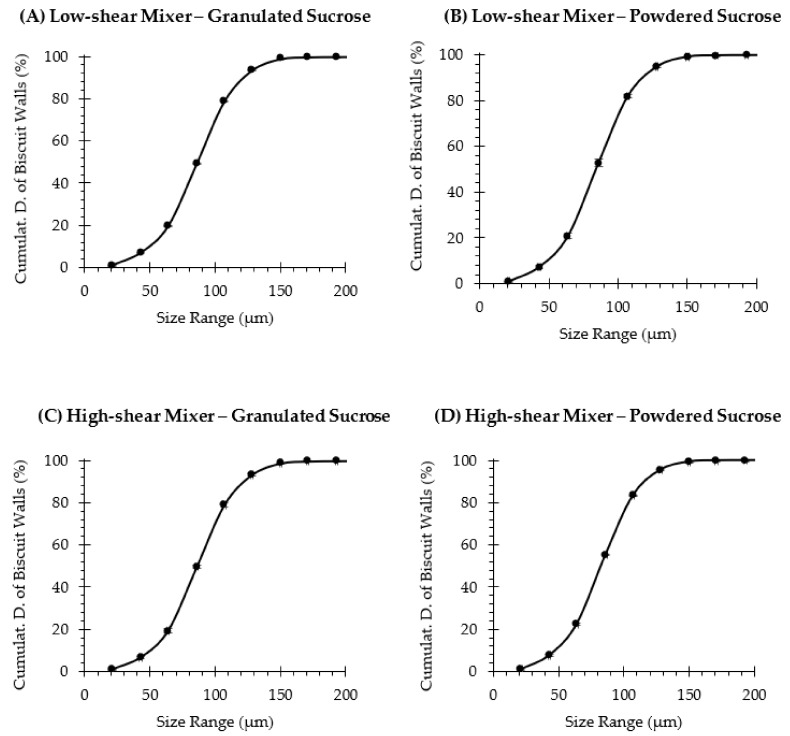
The structure thickness cumulative distribution of the biscuits’ walls, which were elaborated with a sugar solution at 0%ST, using granulated (**A**,**C**) or powdered (**B**,**D**) sucrose, and the creaming phase was subjected to low-shear (**A**,**B**) or high-shear (**C**,**D**) mixing. Data are means ± standard deviation (*n* = 3).

Furthermore, the wall thickness distribution of the biscuit matrix was quantified (3D quantification) also through micro-CT image analysis, as shown in Figure 8. It can be observed that the thickness of biscuit walls is within the range of 21 and 200 µm, and no differences were obtained among the cumulative distributions of samples. For instance, the D_50_ and D_80_ were 86.4 ± 0.1 and 108.8 ± 0.2 for biscuits formulated with granulated sugar and a stable creaming phase (Figure 8A), 84.0 ± 1.0 and 105.6 ± 0.6 for biscuits elaborated with powdered sugar and an unstable creaming phase (Figure 8B), 86.2 ± 0.7 and 108.6 ± 1.5 for biscuits elaborated with granulated sugar and a stable creaming phase by high shearing (Figure 8C) and 82.5 ± 0.3 and 104.5 ± 0.2 for biscuits prepared with powdered sugar and a stable creaming phase by high shearing (Figure 8D). These results show that the stability of the creaming phase did not modify the thickness distribution of the inner walls in rotary-molded biscuits, which could explain the similar results obtained when analyzing the texture and the remaining sensory attributes.

## 4. Conclusions

This study examined the effect of creaming stability on the quality attributes of rotary-molded biscuits. Results show that creaming was unstable when it was produced using a low-shear mixer, where the sucrose particle size and saturated or supersaturated sugar solution also influenced the aqueous-phase migration. However, the use of a high-shear mixer allowed a stable creaming phase to be obtained. Despite the variation in creaming stability, no significant differences were observed in the sensory profile (aeration, noise intensity, grittiness, sweetness and color) of rotary-molded biscuits. In addition, the variation in biscuit dimensions was either inexistent or negligible. The micro-CT analysis revealed that biscuits prepared with an unstable or a stable creaming phase had a similar air porosity and thickness of the biscuit walls. Until now, it was generally accepted that the creaming phase of short doughs had to be stable and emulsified in order to obtain an adequate biscuit. This study suggests that the stability of the creaming phase does not seem to be a relevant factor to determine the quality attributes of rotary-molded biscuits, using the formulations and conditions studied herein.

## Figures and Tables

**Table 1:** Definitions of sensory attributes analyzed by the trainee panel.

Attribute	Definitions
Aeration	Visible air pores at the cross-section of the biscuit.
Color	Level of roasted color at the surface of the biscuit.
Grittiness	Perception of sugar crystals between the tongue and palate or the tongue and teeth.
Hardness	Force required to break the biscuit after the first bite with incisors.
Noise intensity	Overall noise during chewing of biscuit.
Sweetness	Level of perceived sweetness during mastication of biscuit.
Thickness	How thick the biscuit is when looking at its cross-section.

**Table 2:** Dimensions (i.e., length, width, thickness) of rotary-molded biscuits, which were elaborated using granulated or powdered sucrose.

Type of Mixer Type of Sucrose	Sugar Saturation (%ST)	Creaming Stability	Length (mm)	Width (mm)	Thickness (mm)
Low-shear Granulated	0	Stable	39.95 ± 0.14 ^c,e,f^	40.30 ± 0.06 ^g^	5.39 ± 0.04 ^a,c,d,e^
	50	Stable	40.06 ± 0.17 ^c,d,e^	40.27 ± 0.06 ^g,h^	5.23 ± 0.05 ^b^
	100	Unstable	40.26 ± 0.12 ^c,d^	40.35 ± 0.06 ^g^	5.43 ± 0.05 ^c,d,e^
	>100	Unstable	40.42 ± 0.16 ^d^	40.40 ± 0.07 ^g^	5.40 ± 0.05 ^a,c,d,e^
High-shear Granulated	0	Stable	39.53 ± 0.12 ^a,b^	39.88 ± 0.06 ^a,b,c,d^	5.29 ± 0.05 ^a,b^
	50	Stable	39.38 ± 0.17 ^a^	39.88 ± 0.06 ^a,c,d^	5.37 ± 0.04 ^a,c,d,e^
	100	Stable	39.40 ± 0.15 ^a^	39.97 ± 0.07 ^c,d,e^	5.45 ± 0.05 ^c,d,e^
	>100	Stable	40.11 ± 0.14 ^c,d,e^	40.24 ± 0.10 ^f,g,h^	5.48 ± 0.06 ^d,e^
Low-shear Powdered	0	Unstable	40.26 ± 0.13 ^c,d^	40.05 ± 0.06 ^e,f^	5.35 ± 0.04 ^a,c,d^
	50	Unstable	39.96 ± 0.36 ^a–f^	40.04 ± 0.07 ^c,e,f^	5.41 ± 0.06 ^a,c,d,e^
	100	Unstable	39.96 ± 0.12 ^c,e^	40.10 ± 0.08 ^e,f,h^	5.40 ± 0.05 ^a,c,d,e^
	>100	Unstable	40.09 ± 0.14 ^c,d,e^	40.01 ± 0.08 ^c,e^	5.39 ± 0.06 ^a,c,d,e^
High-shear Powdered	0	Stable	39.78 ± 0.15 ^a,b,e,f^	39.76 ± 0.06 ^a,b^	5.35 ± 0.04 ^a,c^
	50	Stable	39.83 ± 0.16 ^b,e,f^	39.79 ± 0.08 ^a,b,d^	5.50 ± 0.06 ^e^
	100	Stable	39.50 ± 0.18 ^a,b^	39.74 ± 0.08 ^a,b^	5.39 ± 0.06 ^a,c,d,e^
	>100	Stable	39.66 ± 0.12 ^a,b,f^	39.72 ± 0.07 ^b^	5.40 ± 0.05 ^a,c,d,e^

Sucrose was dissolved in water to obtain the following sugar solutions: 0%ST, 50%ST, 100%ST or >100%ST. The creaming phase was subjected to low-shear or high-shear mixing. Data of each dimension are means ± confidence intervals at 95%. Different superscripts (a–h) denote significant differences (*p* < 0.05).

**Table 3:** Confidence intervals at 95% of sensory attributes for rotary-molded biscuits.

**Sensory Attributes**	**Aeration**	**Noise**	**Grittiness**	**Sweetness**	**Color**	**Thickness**
**Sugar Saturation (%ST)**	**A: Low-Shear Mixer—Granulated Sucrose**					
0	(2.94, 3.63) ^b^	(4.31, 5.01) ^a^	(1.88, 2.68) ^a^	(5.00, 5.56) ^a,b^	(2.20, 2.92) ^b^	(3.98, 4.43) ^a–c^
50	(2.64, 3.14) ^a,b^	(4.07, 4.92) ^a^	(1.72, 2.55) ^a^	**(5.19, 5.65) ^b^**	(2.17, 2.74) ^b^	(3.86, 4.16) ^a,b^
100	(2.94, 3.45) ^b^	(4.10, 4.88) ^a^	(1.91, 2.77) ^a^	**(4.73, 5.29) ^a^**	(2.02, 2.54) ^a,b^	(3.96, 4.19) ^a–c^
>100	(2.68, 3.37) ^a,b^	(4.23, 5.02) ^a^	(1.69, 2.59) ^a^	(5.08, 5.73) ^a,b^	(2.21, 2.70) ^b^	(3.99, 4.25) ^a–c^
**Sugar Saturation (%ST)**	**B: Low-Shear Mixer—Powdered Sucrose**					
0	(2.55, 3.16) ^a,b^	(4.19, 5.01) ^a^	(1.82, 2.82) ^a^	(5.01, 5.70) ^a,b^	(2.18, 2.60) ^b^	(4.00, 4.26) ^b,c^
50	(2.32, 2.94) ^a^	(4.03, 4.77) ^a^	(2.06, 2.85) ^a^	(4.96, 5.57) ^a,b^	(2.09, 2.65) ^a,b^	(3.80, 4.33) ^a–c^
100	(2.61, 3.18) ^a,b^	(4.22, 5.00) ^a^	(1.89, 2.81) ^a^	(4.74, 5.43) ^a,b^	(2.10, 2.69) ^b^	(4.07, 4.55) ^c^
>100	(2.97, 3.56) ^b^	(4.30, 5.02) ^a^	(1.86, 2.59) ^a^	(5.04, 5.65) ^a,b^	(2.03, 2.51) ^a,b^	(4.01, 4.66) ^b,c^
**Sugar Saturation (%ST)**	**C: High-Shear Mixer—Granulated Sucrose**					
0	(2.73, 3.24) ^a,b^	(4.24, 5.09) ^a^	(1.87, 2.57) ^a^	(5.09, 5.67) ^a,b^	(2.29, 2.67) ^b^	**(3.70, 4.06) ^a^**
50	(2.90, 3.61) ^b^	(4.54, 5.34) ^a^	(1.72, 2.48) ^a^	(4.87, 5.40) ^a,b^	(2.13, 2.68) ^b^	(3.95, 4.28) ^a–c^
100	(2.65, 3.39) ^a,b^	(4.38, 5.11) ^a^	(1.62, 2.25) ^a^	(4.99, 5.55) ^a,b^	(2.02, 2.50) ^a,b^	(3.88, 4.24) ^a–c^
>100	(2.62, 3.18) ^a,b^	(4.29, 5.16) ^a^	(1.62, 2.30) ^a^	(4.89, 5.54) ^a,b^	(2.29, 2.80) ^b^	**(3.97, 4.34) ^b,c^**
**Sugar Saturation (%ST)**	**D: High-Shear Mixer—Powdered Sucrose**					
0	(2.44, 3.20) ^a,b^	(4.44, 5.15) ^a^	(1.67, 2.52) ^a^	(4.81, 5.28) ^a^	(1.92, 2.49) ^a,b^	(3.78, 4.36) ^a–c^
50	(2.67, 3.33) ^a,b^	(4.17, 4.95) ^a^	(1.70, 2.46) ^a^	(4.94, 5.51) ^a,b^	**(2.17, 2.49) ^b^**	(4.09, 4.56) ^c^
100	(2.46, 3.25) ^a,b^	(4.33, 5.19) ^a^	(1.95, 2.81) ^a^	(4.83, 5.36) ^a,b^	(2.02, 2.50) ^a,b^	(3.83, 4.42) ^a–c^
>100	(2.84, 3.32) ^a,b^	(4.51, 5.22) ^a^	(1.68, 2.45) ^a^	(4.84, 5.41) ^a,b^	**(1.68, 2.25) ^a^**	(3.97, 4.36) ^a–c^

Biscuits were elaborated using granulated or powdered sucrose. Sugar was dissolved in water to obtain the following sugar solutions: 0%ST, 50%ST, 100%ST or >100%ST. Further, the creaming phase was subjected to low-shear or high-shear mixing. Different superscripts per attribute denote significant differences (*p* < 0.05). Bolded values indicate significant differences at different sugar solutions.

## Data Availability

Data are contained within the article.
